# Public Attitudes Toward Violence Against Doctors: Sentiment Analysis of Chinese Users

**DOI:** 10.2196/63772

**Published:** 2025-03-20

**Authors:** Yuwen Zheng, Meirong Tian, Jingjing Chen, Lei Zhang, Jia Gao, Xiang Li, Jin Wen, Xing Qu

**Affiliations:** 1 Institute of Hospital Management West China Hospital Sichuan University Chengdu China

**Keywords:** doctor-patient conflict, sentiment analysis, latent Dirichlet allocation, LDA, social media analysis, public health crisis

## Abstract

**Background:**

Violence against doctors attracts the public’s attention both online and in the real world. Understanding how public sentiment evolves during such crises is essential for developing strategies to manage emotions and rebuild trust.

**Objective:**

This study aims to quantify the difference in public sentiment based on the public opinion life cycle theory and describe how public sentiment evolved during a high-profile crisis involving violence against doctors in China.

**Methods:**

This study used the term frequency-inverse document frequency (TF-IDF) algorithm to extract key terms and create keyword clouds from textual comments. The latent Dirichlet allocation (LDA) topic model was used to analyze the thematic trends and shifts within public sentiment. The integrated Chinese Sentiment Lexicon was used to analyze sentiment trajectories in the collected data.

**Results:**

A total of 12,775 valid comments were collected on Sina Weibo about public opinion related to a doctor-patient conflict. Thematic and sentiment analyses showed that the public’s sentiments were highly negative during the outbreak period (disgust: 10,201/30,433, 33.52%; anger: 6792/30,433, 22.32%) then smoothly changed to positive and negative during the spread period (sorrow: 2952/8569, 34.45%; joy: 2782/8569, 32.47%) and tended to be rational and peaceful during the decline period (joy: 4757/14,543, 32.71%; sorrow: 4070/14,543, 27.99%). However, no matter how emotions changed, each period's leading tone contained many negative sentiments.

**Conclusions:**

This study simultaneously examined the dynamics of theme change and sentiment evolution in crises involving violence against doctors. It discovered that public sentiment evolved alongside thematic changes, with the dominant negative tone from the initial stage persisting throughout. This finding, distinguished from prior research, underscores the lasting influence of early public sentiment. The results offer valuable insights for medical institutions and authorities, suggesting the need for tailored risk communication strategies responsive to the evolving themes and sentiments at different stages of a crisis.

## Introduction

The rising incidence of doctor-patient conflicts has become a significant concern globally. The Global Health Workforce Statistics database of the World Health Organization (WHO) highlights an increasing trend in medical disputes driven by inequitable distribution of medical resources and heightened pressures on health care systems [1]. The WHO’s “Global Strategy on Human Resources for Health: Workforce 2030” emphasizes the importance of equitable access to health care workers and robust health systems to address and prevent doctor-patient disputes [2].

Doctor-patient conflicts, broadly defined as disputes or tensions between medical professionals and patients (or their families), arise from dissatisfaction with medical outcomes, communication breakdowns, or systemic health care challenges. These conflicts vary in intensity, ranging from verbal disputes to severe incidents of physical harm. Violence against doctors represents the most extreme form of these conflicts, involving verbal abuse, threats, or physical assaults that jeopardize the safety and well-being of health care providers while eroding trust in the medical system [3].

In China, doctor-patient conflicts have been shaped by unique cultural, social, and structural factors [4]. Surveys conducted by the Chinese Medical Doctor Association reported that over 60% of physicians experience verbal or physical conflicts with patients during their careers [5], reflecting pervasive tension in medical interactions. These conflicts are often exacerbated by high patient expectations and increased access to medical information, which amplify scrutiny of health care practices. Structural challenges in the Chinese health care system also contribute significantly. According to the National Health Commission of China, urban hospitals handle over 3 billion outpatient visits annually, resulting in overcrowded facilities and excessive workloads for medical staff [6]. These systemic pressures often lead to long waiting times and dissatisfaction, which can escalate into disputes. Given the critical nature of these issues, there is a pressing need for systematic research on doctor-patient conflicts. Analyzing specific incidents of such conflicts can provide valuable insights into their social, psychological, and institutional determinants. Effective research and targeted interventions can enhance communication, improve patient satisfaction, and ensure patients’ and medical professionals’ safety and well-being, ultimately contributing to a more efficient and humane health care system.

In health communication, understanding the different developmental stages of events is crucial for designing and implementing effective public relations strategies [7,8]. Analyzing health events from a temporal dimension can reveal the patterns of changes in public sentiment and attention throughout various stages, including incubation, outbreak, spread, and resolution. Furthermore, integrating temporal analysis with topic and sentiment dimensions offers a more comprehensive understanding of the effectiveness of health information dissemination [9].

In medical public opinion research, theme analysis is equally crucial as segmenting the development stages of public sentiment. It is a widely used method in academia [10,11]. Theme analysis involves profound interpretation of textual data to reveal underlying meanings and critical issues that capture public interest, thus helping researchers accurately identify the focal points of public attention. Additionally, through systematic data processing, theme analysis provides a dynamic view of changes in public opinion, aiding researchers with understanding the fundamental shifts in public responses to health information.

Although theme analysis can reveal topics of public interest, more is needed to comprehensively understand the complexity of public attitudes. Consequently, in recent years, the academic community has emphasized sentiment analysis to explore the public’s deep emotional tendencies and responses to public opinion [12-14]. Sentiment analysis methods include deep learning, machine learning, and lexicon-based analysis. The lexicon-based approach has unique advantages when dealing with structured and unstructured text data. Compared with deep learning and machine learning methods, lexicon-based analysis relies on a predefined set of sentiment words, providing more intuitive and interpretable results [15]. When applied to sentiment analysis in specific domains, such as doctor-patient conflicts, this method can effectively capture the emotional nuances in professional terminology and industry-specific language, thereby ensuring the accuracy and relevance of the analysis [16,17].

The occurrence of doctor-patient conflicts might be associated with the unequal distribution of medical resources [18], patient expectations and satisfaction [19], and communication skills between doctors and patients [20]. In 2021, an assault incident at Beijing Chaoyang Hospital garnered prolonged and extensive societal attention. During the incidents, Dr. Tao Yong, an ophthalmologist, sustained severe injuries to his head and arms, which put his life in jeopardy and subsequently impaired his ability to conduct surgeries postrecovery. The public response to this incident was significantly more intense than other medical disputes during the same period, indicating its profound impact on public sentiment and medical community relations.

This study, based on the theoretical perspective of the online public opinion life cycle, used the latent Dirichlet allocation (LDA) topic model and sentiment lexicon from Dalian University of Technology to conduct topic and sentiment analysis on the “Beijing Chaoyang Hospital assault incident,” a significant doctor-patient conflict incident in China. The aim was to fill the research gap in medical public opinion and assist medical institutions and government departments to better understand the direction of public opinion dissemination and emotional contagion pathways in related events. By developing targeted content publishing and sentiment guidance strategies in response to doctor-patient conflict incidents, the study sought to improve communication effectiveness and ensure risk management and response efficiency.

## Methods

### Methodological Framework

This study divided opinion stages based on the life cycle of public opinion, which is often applied to the survey of public sentiment events. Life cycle theory, as a broadly adopted theoretical perspective, provides scholars with a structured framework to analyze the stages of change and development in events. Life cycle theory provides a systematic approach to dividing the public opinion cycle into stages, ensuring a theoretically grounded methodology for data segmentation and thematic analysis [21]. The life cycle theory, proposed by Steven Fink [22], posited that crises evolve through 4 phases: incubation, outbreak, spread, and resolution. Similarly, the life cycle of online public sentiment begins with initial attention, escalates with event progression, and declines after saturation, mirroring societal public sentiment development [23-26]. This study followed this theoretical approach to segment public opinion into the outbreak, spread, and decline phases, aligning with previous applications in public discourse analysis [27,28].

Data were collected from Sina Weibo, the most widely used social media platform in China, akin to X (previously known as Twitter). According to the China Internet Network Information Center 2022 report, Sina Weibo had over 582 million monthly active users [29], making it a primary platform for public discourse and sentiment expression in China. The platform has been widely used in studies of Chinese public opinion, serving as a critical data source for analyzing sentiment and social behavior [30].

**Figure 1:**
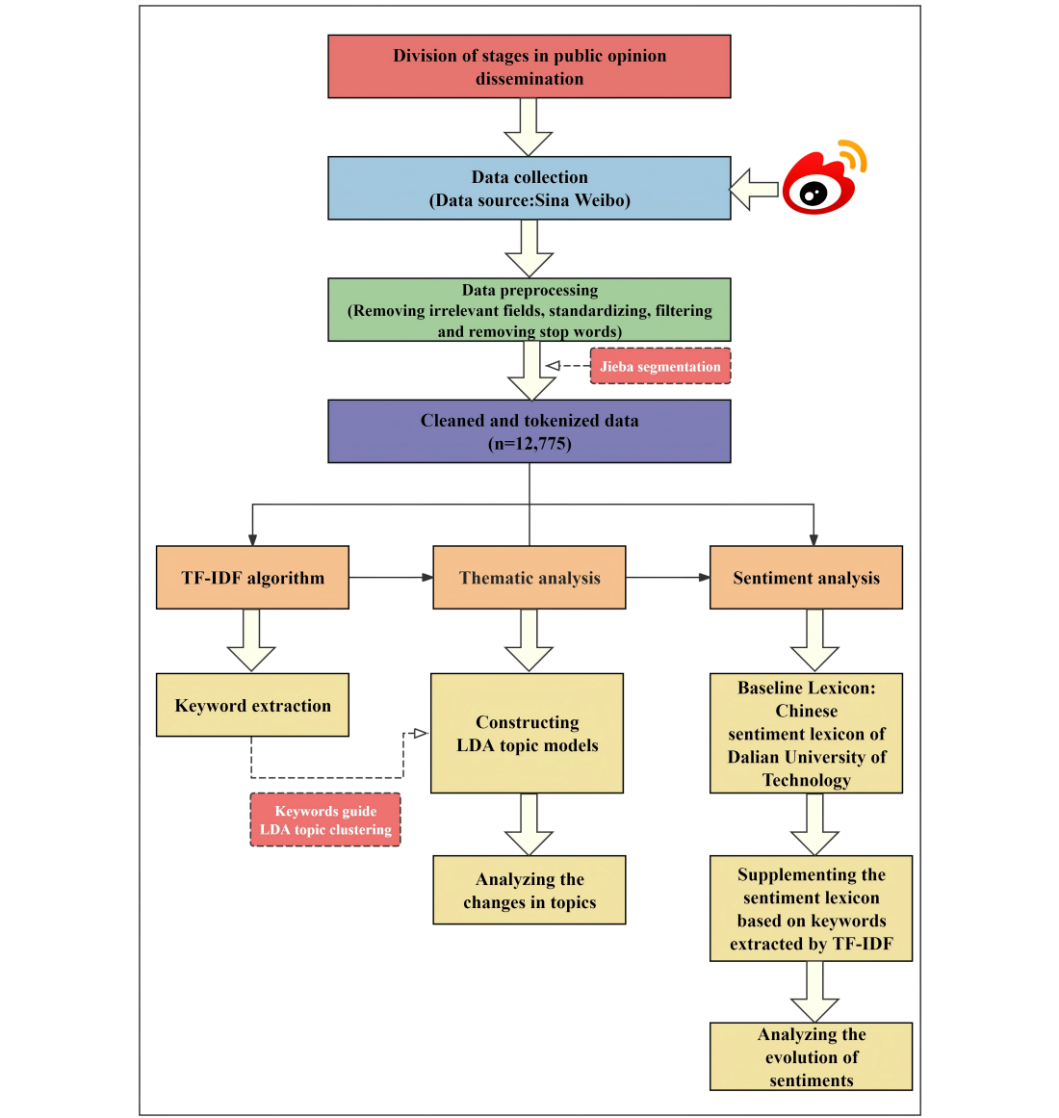
The methodological framework of the proposed research. LDA: latent Dirichlet allocation; TF-IDF: term frequency-inverse document frequency.

We collected relevant comment data from Sina Weibo according to the public sentiment development cycle. After cleaning and segmenting the data, the term frequency-inverse document frequency (TF-IDF) algorithm was used to extract keywords, and the LDA topic model was used to analyze the focal points across different stages. Finally, a sentiment lexicon was constructed using the Chinese Sentiment Lexicon of the Dalian University of Technology (DUTIR), and sentiment analysis was conducted based on this lexicon. Figure 1 illustrates the methodological framework.

### Public Sentiment Dissemination Stage Division

**Figure 2:**
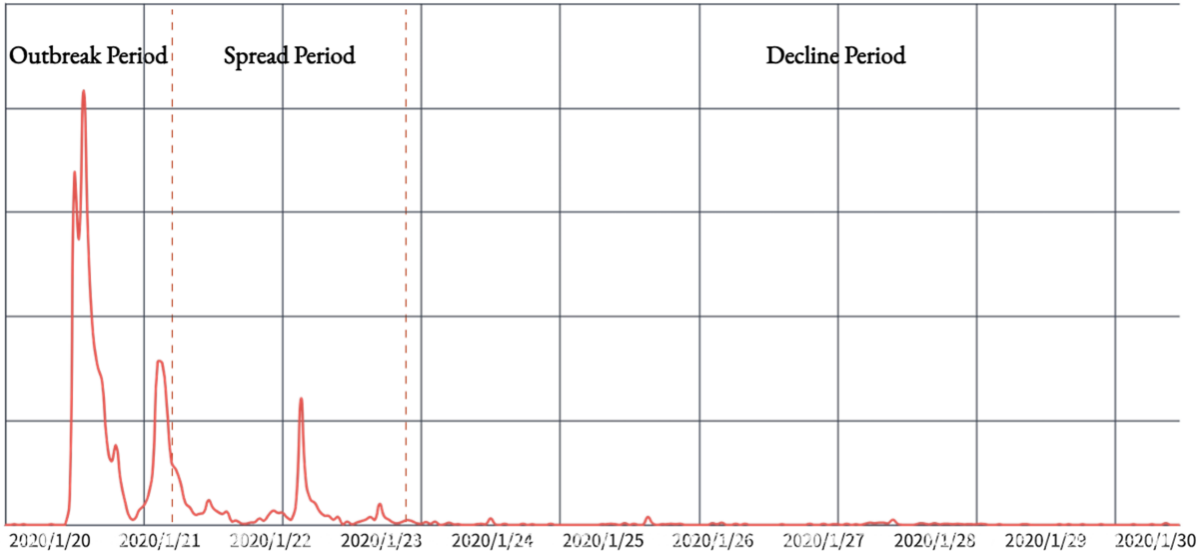
Public opinion life cycle division of the “Beijing Chaoyang Hospital assault incident”.

This study analyzed the trend of public opinion on the website Zhiwei Data. The trends were divided into 3 development periods, as illustrated in Figure 2. Zhiwei Data is a platform that monitors and analyzes China’s internet big data. The platform uses social media data from across the internet to depict the development trends of events, communication effects, and trends in social communication.

### Data Collection

The research data were sourced from Sina Weibo, China’s largest social media platform mirroring X’s functionality and reach. It boasts a broad user base and spans various topics, providing a valuable snapshot of public discourse and sentiment. This study, based on the development of public opinion and the 3 life cycles of public opinion, selected 11 hashtags generated during the public opinion period of the “Beijing Chaoyang Hospital assault incident” in chronological order, with the “#The ophthalmologist at Chaoyang Hospital was hacked on 20 January 2020” as the starting point and the “#Dr. Tao Yong resumed clinic visits on 13 May” as the termination point to disseminate public opinion.

To collect comments related to these hashtags, the Python library Scrapy was used. Data collection occurred over a 4-week period, ensuring the capture of both initial reactions and ongoing public discourse. A total of 12,775 valid comments were collected after cleaning and filtering.

### Data Preprocessing

Data preprocessing is a crucial step to ensure the consistency, accuracy, and relevance of the data used for sentiment analysis. The following subsections describe the key steps involved in preparing the data for analysis.

#### Data Cleaning

This stage involved removing invalid fields, standardizing data formats, and filtering out irrelevant content.

To remove duplicates, we identified and removed duplicate entries based on text, time stamps, and user IDs. This step reduced the data set by approximately 1200 comments.

To filter irrelevant content, we eliminated promotional, spam, or off-topic comments through keyword-based filtering and manual review. Approximately 500 comments were removed.

To eliminate invalid fields, we excluded records with incomplete or corrupted data fields, removing 500 comments.

After cleaning, the data set was reduced from 15,000 comments to 12,800 comments for further processing.

#### Stop Word Filtering

Stop words were identified and removed based on a predefined list derived from linguistic resources. These typically include high-frequency function words such as prepositions, conjunctions, and pronouns that do not contribute to the semantic content of the text. This process is consistent with best practices in sentiment analysis [27]. The stop word list was further refined by reviewing a sample of the data set to identify any frequently occurring terms that, although noninformative in general contexts, might carry domain-specific relevance (eg, medical terms) and should thus be retained. As a result, irrelevant terms such as “the,” “and,” and “of” were removed, while domain-relevant but functionally noninformative terms (eg, “patient,” “doctor”) were retained, following similar practices in health care sentiment analysis [31-33].

#### Lexicon Refinement

The word segmentation process was carried out using the Jieba tool, which uses a domain-specific lexicon tailored to the study’s needs. This lexicon was iteratively refined based on the segmentation results. Initially populated with general terms, the lexicon was updated by adding domain-specific terminology, particularly medical and health care–related terms, to enhance segmentation accuracy. For example, terms such as “doctor-patient relationship” and “medical malpractice” were incorporated to ensure that multiword expressions were correctly recognized. The lexicon was reviewed and updated after each iteration, ensuring it aligned with the research requirements and was capable of handling both general and specialized terms essential for analyzing public sentiment regarding doctor-patient conflicts [34].

#### Text Segmentation

Text segmentation was conducted using the Jieba tool, which splits text into individual words for further analysis. The lexicon refined in the previous step ensured that domain-specific terms were correctly segmented. The segmentation results were evaluated and adjusted manually to guarantee that critical multiword expressions were preserved and that the segmentation was contextually appropriate for the health care domain. This process was crucial in ensuring that the segmented words accurately reflected the topics discussed in the comments, particularly in the context of doctor-patient conflicts.

#### Final Data Volume

After preprocessing—including cleaning, stop word removal, lexicon refinement, and segmentation—the final data set comprised 12,775 valid and tokenized comments, as indicated in Figure 1.

#### TF-IDF

TF-IDF is a commonly used algorithm in text mining and information retrieval to measure the significance of a word in a text. The algorithm combines 2 crucial factors: term frequency and inverse document frequency. It calculates the frequency of each word in the text and its inverse document frequency in the corpus. It then assigns a weight to each word to reflect its importance in the collection of texts [35].

TF-IDF has been widely applied in various domains, including health care, where it is used to identify key terms in patient feedback, online health forums, and social media platforms. For example, research has demonstrated effectiveness in uncovering critical discussion themes in public health and medical services. Studies have used TF-IDF to analyze patient complaints and feedback to identify key areas for service improvement [36] and to extract critical health-related terms from social media during health crises such as the COVID-19 pandemic [37]. However, its application in analyzing the doctor-patient relationship remains limited, representing a gap this study aimed to address. In this study, TF-IDF was used to extract keywords from the Sina Weibo data set, ensuring the identification of terms most relevant to public discourse on doctor-patient conflicts.

TF represents the frequency of a word in the text. It is calculated as follows:



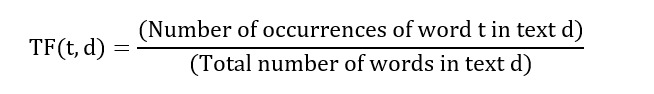



IDF represents the inverse document frequency of a word in the entire corpus. It is calculated as follows:



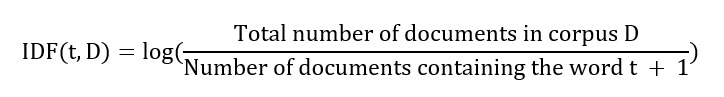



The TF-IDF weights are ultimately formed by the product of TF and IDF:

TF–IDF(t,d,D)=TF(t,d) × IDF(t,D)

#### LDA Topic Model

LDA is a probabilistic graphical model to identify hidden thematic structures within text collections. The model was proposed by Blei et al [38] in 2003 and has become a classic method for topic modeling in text mining. The LDA model extracts potential topics from documents or corpora using a bag-of-words approach. It constructs a model that includes a “document-topic distribution” and “topic-word distribution” without considering the order in which words appear [39]. The document comprises one or more topics, with each word generated by one of the topics [40]. Therefore, LDA models can aid in text-based analysis processes for online opinion topic events in social media, such as identifying latent topics and clustering users. In the field of health communication, the LDA model has been widely applied to identify themes related to sudden public health events, such as COVID-19 [41,42].

**Figure 3:**
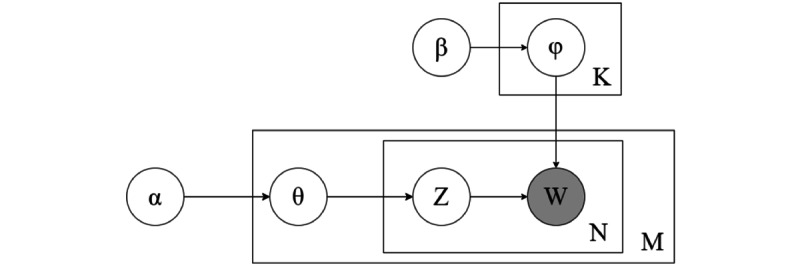
The generation process of the latent Dirichlet allocation (LDA) topic model.

The generation process of the LDA topic model is shown in Figure 3: (1) generate the hyperparameter α of the topic distribution and generate the topic distribution θ for each document; (2) for each document, generate the topic Z from its topic distribution θ; (3) for each topic K, generate the word distribution φ of the topic K from the hyperparameter β of the word distribution; (4) for the Nth word in each document, generate it from the word distribution of its corresponding topic Z; (5) W represents the words in the corpus, which are generated based on their respective topic-word distributions (φ); (6) N is the number of words in a document, representing the scope of word generation within each document; and (7) M is the total number of documents in the data set, defining the corpus-level scope of the generative process.

#### Sentiment Lexicon

Sentiment analysis and topic modeling have been widely applied as effective tools to analyze public sentiment and identify thematic patterns in large-scale social media data sets [43]. It is first necessary to construct a lexicon of sentiments to dynamically analyze public opinion sentiments and themes in doctor-patient conflict cases. This study used the DUTIR, which was developed by the Information Retrieval Research Laboratory based on the Chinese language context, as the basic sentiment lexicon. It is used for sentiment analysis and sentiment calculation to help analyze the sentiment tendency in texts and is widely used in the field of Chinese text research as a whole. This lexicon has been used in various research studies, demonstrating its reliability and applicability in different sentiment analysis scenarios. In health care, sentiment lexicons have been extensively used to assess patient satisfaction [44], analyze public sentiment during health crises [45], and evaluate feedback on health care services [46]. Research has shown that DUTIR effectively captures nuanced emotional expressions in Chinese texts, such as in sentiment-aware word embedding for emotion classification. Additionally, DUTIR has proven valuable in analyzing sentiment trends in online media, including bullet screen comments on health care topics. Most existing sentiment analysis research in health care classified collected comment texts into binary categories, simply dividing public sentiment into positive and negative [47-49], which failed to vividly capture the diversity of public emotions. The sentiment lexicon used in this study introduced neutral, complex, and mixed emotional categories, allowing for a more refined capture of the comment texts and reflecting the multidimensional characteristics of emotions. The lexicon contains 27,466 sentiment words, and the sentiment categories are divided into 7 types: joy, happiness, anger, disgust, fear, sorrow, and surprise. The selection of these 7 categories is primarily rooted in the predefined structure of DUTIR, which has been validated in previous studies and widely applied in public discourse and health care research. Research has shown that DUTIR’s categorization effectively captures emotional nuances, making it particularly suited for complex sentiment analysis tasks. Its application in health care sentiment analysis and public discourse has been widely documented [31,50], underscoring its relevance and reliability.

Additionally, the chosen classifications align with established psychological theories, such as the model of universal emotions by Ekman and Friesen [51] and wheel of emotions by Plutchik [52]. These frameworks identify emotions such as joy, anger, and fear as universal and fundamental, providing a robust theoretical basis for the classification system. This dual grounding in theoretical models and the practical structure of DUTIR ensures that the framework is both comprehensive and contextually relevant.

By leveraging the predefined structure of DUTIR and its theoretical underpinnings, this study effectively captured the multidimensional emotional dynamics in public sentiment surrounding doctor-patient conflicts, offering insights beyond binary sentiment classifications.

### Ethical Considerations

This study involved the analysis of publicly available, anonymized data from Sina Weibo. Since no personally identifiable information was collected, stored, or analyzed, and no direct interaction with individuals occurred, the study was exempt from Institutional Review Board (IRB) approval in accordance with Sichuan University's Research Ethics Review Policy.

Informed consent was not required, as this study relied exclusively on publicly available user-generated content. Additionally, the study adhered to the terms of service and privacy policies of the Sina Weibo platform.

## Results

### TF-IDF Keyword Extraction

The study conducted TF-IDF keyword extraction to identify the primary words associated with the “Beijing Chaoyang Hospital assault incident” at different public opinion stages. During the outbreak period, the most frequently expressed words were “injury” (TF-IDF: 0.030589), “doctor” (TF-IDF: 0.028905), and “harm” (TF-IDF: 0.028270). In the spread period, “gentle” (TF-IDF: 0.05255), “blessings” (TF-IDF: 0.05179), and “kindness” (TF-IDF: 0.05060) surfaced frequently. By the decline period, the words “recovery” (TF-IDF: 0.050599), “injury” (TF-IDF: 0.047823), and “gentle” (TF-IDF: 0.047587) were most prevalent.

Table 1 summarizes the high-frequency keywords and their corresponding frequencies and TF-IDF values for the 3 periods, illustrating the changes in public focal points and high-frequency expressions at each stage.

In the outbreak period, the words were dominated by emotionally charged terms like “harm to doctors,” “injury,” and “angry to death,” reflecting public shock and outrage. During the spread period, supportive and empathetic words such as “blessings,” “gentle,” and “recovery” emerged, signifying a shift toward collective concern and healing. By the decline period, the focus remained on resolution with terms like “recovery,” “criminal,” and “hatred,” suggesting ongoing calls for justice alongside a desire for closure.

**Table 1:** Term frequency-inverse document frequency (TF-IDF) keyword extraction for 3 stages of public opinion.

Word		Frequency	TF-IDF
**Outbreak period**			
	Injury	1465	0.030589
	Doctor harm	942	0.028905
	Harm	792	0.028270
	Prayer	629	0.027485
	Medical study	612	0.027354
	Enrage	671	0.027305
	Doctor consult	561	0.026949
	Security check	551	0.026922
	Excellent	533	0.026804
	Treatment	522	0.026768
**Spread period**			
	Gentle	386	0.052555
	Blessings	349	0.051793
	Kindness	302	0.050600
	Recovery	238	0.048901
	Heartbroken	186	0.047038
	World	182	0.045853
	Brightness	157	0.045260
	Safety	167	0.045209
	Excellent	145	0.045105
	Good person	144	0.044783
**Decline period**			
	Recovery	865	0.050599
	Injury	513	0.047823
	Gentle	495	0.047587
	Hatred	402	0.046213
	Recovery	382	0.045876
	Culprit	589	0.045392
	Encourage	354	0.045373
	Hospital discharge	283	0.043894
	Wish	280	0.043800
	Harm	249	0.042751

### LDA Topic Model

**Figure 4:**
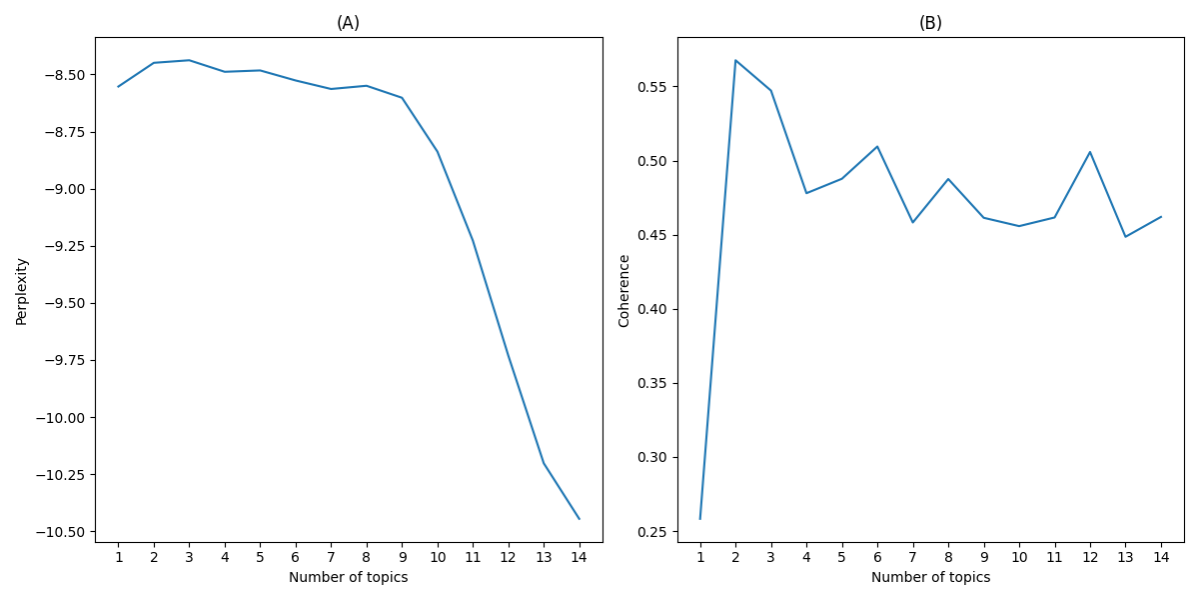
(A) Perplexity and (B) coherence scores of latent Dirichlet allocation (LDA) topics during the outbreak period.

**Figure 5:**
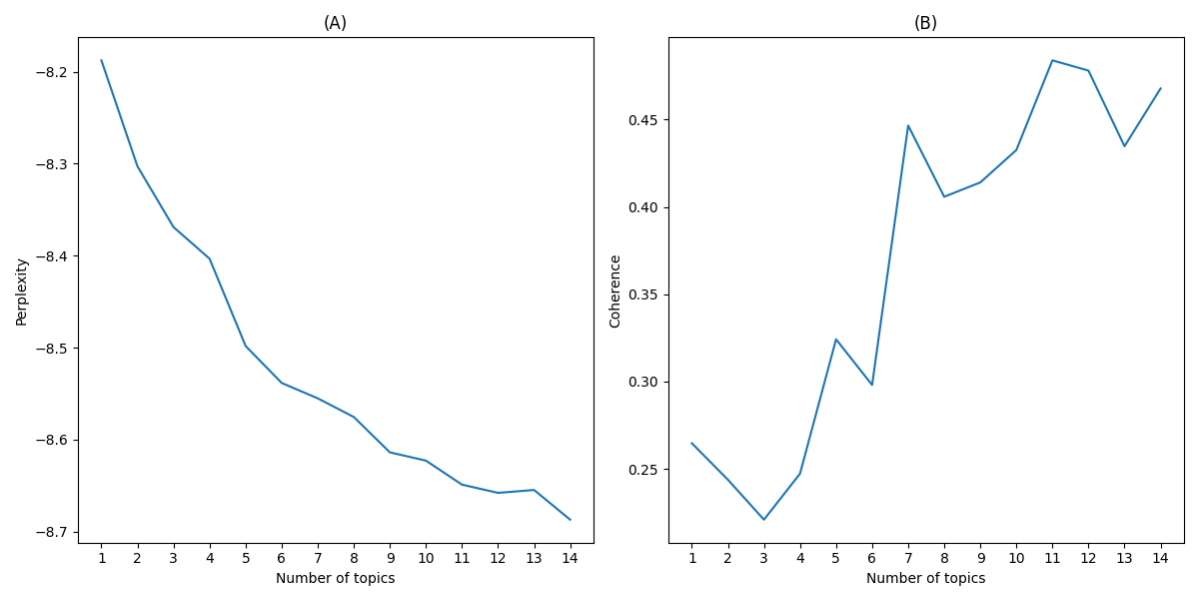
(A) Perplexity and (B) coherence scores of latent Dirichlet allocation (LDA) topics during the spread period.

**Figure 6:**
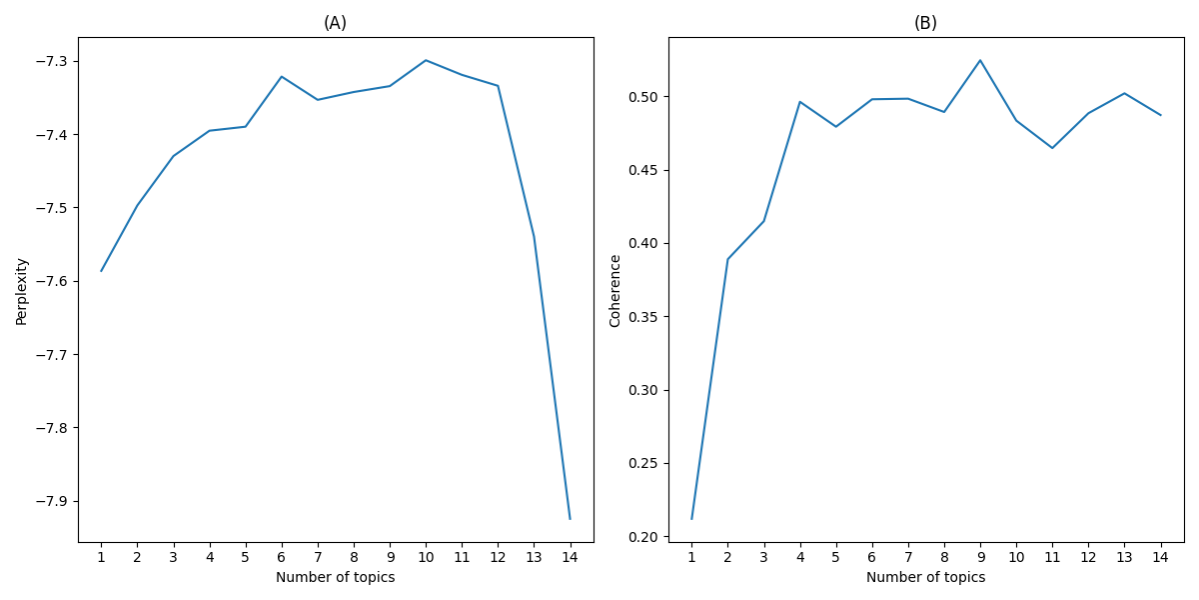
(A) Perplexity and (B) coherence scores of latent Dirichlet allocation (LDA) topics during the decline period.

Using the Gensim library in Python for LDA topic model training, the study focused on perplexity and coherence to gauge model quality and thematic clarity. Perplexity was used to measure the model’s ability to predict the test data, with lower perplexity indicating better model performance [38]. Coherence was used to assess the interpretability of the topics, with higher coherence scores reflecting clearer and more meaningful issues [53]. Evaluating these metrics under different settings revealed the optimal theme count. Figures 4-6 illustrate these metrics across public opinion dynamics stages—outbreak, spread, and decline—aiding with precise determination of topic numbers in sync with data volume for effective topic identification and extraction.

To enhance the coherence and interpretability of the LDA results, TF-IDF–extracted keywords were used as seed words to guide topic analysis [54]. High-weighted TF-IDF terms ensured that LDA-generated topics were aligned with public discourse at each stage. For instance, during the outbreak period, keywords like “injury,” “doctor,” and “harm” shaped topics centered on medical accountability and incident responses. In the spread phase, terms such as “gentle” and “blessings” informed themes of empathy and community healing, while “recovery” and “criminal” in the decline period contributed to topics related to resolution and justice. This integration ensured that the LDA analysis was both data-driven and contextually grounded, aligning topic clusters with the most critical public concerns.

As illustrated in Figure 4, the perplexity score steadily declined with an increasing number of topics, while the coherence score peaked at 11 topics. This peak indicated that 11 topics provided the most meaningful representation of initial public reactions, capturing themes such as outrage, shock, and immediate demands for justice.

During the spread phase, as depicted in Figure 5, the coherence score reached its maximum value at 12 topics, while the perplexity curve exhibited diminishing returns beyond this point. This selection reflected the complexity of public discussions during this phase, including sympathy for medical professionals, criticism of systemic health care issues, and debates on policy implications.

Figure 6 shows that coherence peaked at 13 topics during the decline phase, while the perplexity score stabilized. This topic number aligned with the reflective and solution-oriented nature of public discourse in this period, encompassing themes such as health care reforms, institutional accountability, and the long-term impacts of the incident.

After determining the optimal number of topics, we used the pyLDAvis library in Python to create an interactive visualization of the topic model. The study then selected 10 higher-weight keywords to annotate and analyze the topics. The results showed that, during the outbreak period, topics centered on immediate responses to medical violence (Topic 1), victim empathy (Topic 2), societal disillusionment (Topic 3), aggressor accountability (Topic 4), and recovery aspirations (Topic 5). As the incident progressed, the public focus shifted toward appreciating the victim’s character (Topic 1), supporting their recovery (Topic 2), expressing concerns over the career impact (Topic 3), condemning the aggression (Topic 4), and expressing relief over the victim’s recovery (Topic 5). In the decline phase, discussions centered on the patient’s condition (Topic 1), the victim’s virtues (Topic 2), well wishes for recovery (Topic 3), strategies for resolving medical disputes (Topic 4), and advocacy for stringent legal measures (Topic 5). This comprehensive thematic analysis highlighted the dynamic evolution of public sentiment and concerns throughout the incident’s life cycle. The complete LDA topic extraction is presented in Table 2.

**Table 2:** Latent Dirichlet allocation (LDA) topic identifiers for 3 periods of public opinion.

Life cycle period topics			Terms
**Outbreak period**			
	Topic 1: Responses to Medical Violence	Enraged; security check; medical injury; medical disturbance; busy; injury; society; life; slash; harm	
	Topic 2: Victim Empathy	Effort; society; excellent; recovery; medical student; save lives; cry; cultivate; good person; contribute	
	Topic 3: Societal Disillusionment	China; study medicine; can’t save; Lu Xun; disheartened; statement; epidemic; guard; darkness; difficult	
	Topic 4: Aggressor Accountability	Protection; death penalty; severe punishment; medical; country; murderer; law; medical disturbance; aggressor; expose	
	Topic 5: Recovery Aspirations	Pray; safety; heartache; sad; excellent; speedy recovery; light; medical disturbance; injury; destroy	
**Spread period**			
	Topic 1: Victim Character Appreciation	Sad; excellent; gentle; angel; harm; kind; heartache; self; death penalty; good person	
	Topic 2: Recovery Support	Cry; pay tribute; pray; save; care; indispensable; cheer up; heartache; speedy recovery; light	
	Topic 3: Career Impact Concerns	Heartache; pity; excellent; kind; top; recovery; unable; research; scientific research; cultivate	
	Topic 4: Condemnation of Aggression	Harm; furious; harsh punishment; strict punishment for murderers; heartache; destroy; medical disturbance; undeserving; sad; angry	
	Topic 5: Relief Over Victim’s Awakening	Bless; miracle; safety; wake up; good news; take care; thankfully; hope; God; fortunately	
**Decline period**			
	Topic 1: Patient Condition Focus	Body; treatment; recovery; nerve; rupture; blood vessel; muscle; Gate of Hell; mental state; perception	
	Topic 2: Victim Virtue Highlight	Gentle; forgive; kind; benevolence; speedy recovery; strong; inner; moved; beautiful; blessings	
	Topic 3: Well-Wishes for the Victim	Wish; safety; good person; speedy recovery; gentle; heartache; angel; safe and sound; healthy; memory	
	Topic 4: Strategies for Medical Dispute Resolution	Doctor-patient; protection; doctor-patient relationship; policy; medical; respect; country; inspection; medical disturbance; security check	
	Topic 5: Advocacy for Stringent Legal Measures	Recovery; apology; harsh punishment for murderers; law; harm; cost; doctor-patient conflict; death penalty; medical injury; injure	

### Sentiment Analysis

#### Construction of Sentiment Lexicon

Owing to changes in language and the online environment, the connotations of certain words and sentences shifted, and new vocabulary emerged. Therefore, this study expanded the existing DUTIR by manually annotating the emotional characteristics of new words according to the ontology format of the existing database. This was done to cater to the demand for sentiment analysis in specific events or generic scenarios and to ensure accurate results. Multiple annotators working together to extract vocabulary is a recognized research method in sentiment analysis, as it helps improve reliability and reduce bias in the annotation process [55,56]. The expansion process involved several steps to maintain consistency and reliability. First, new words were identified based on their frequency and contextual relevance to the doctor-patient conflict data set. The emotional characteristics of these words were then annotated manually by multiple scholars. Each scholar independently assigned emotional labels to the words, following the ontology structure of the existing lexicon.

Discrepancies between annotations were resolved through discussion and consensus-building among the scholars. Finally, the updated lexicon was validated against a subset of the data to ensure alignment with the existing lexicon’s structure and objectives. The study compared the keywords extracted by the TF-IDF algorithm with the sentiment vocabulary list of the Dalian University of Technology. We selected the keywords not included in the sentiment vocabulary ontology database and manually added them after labeling them with emotional features. This process formed an expanded sentiment lexicon.

#### Sentimental Trend

**Figure 7:**
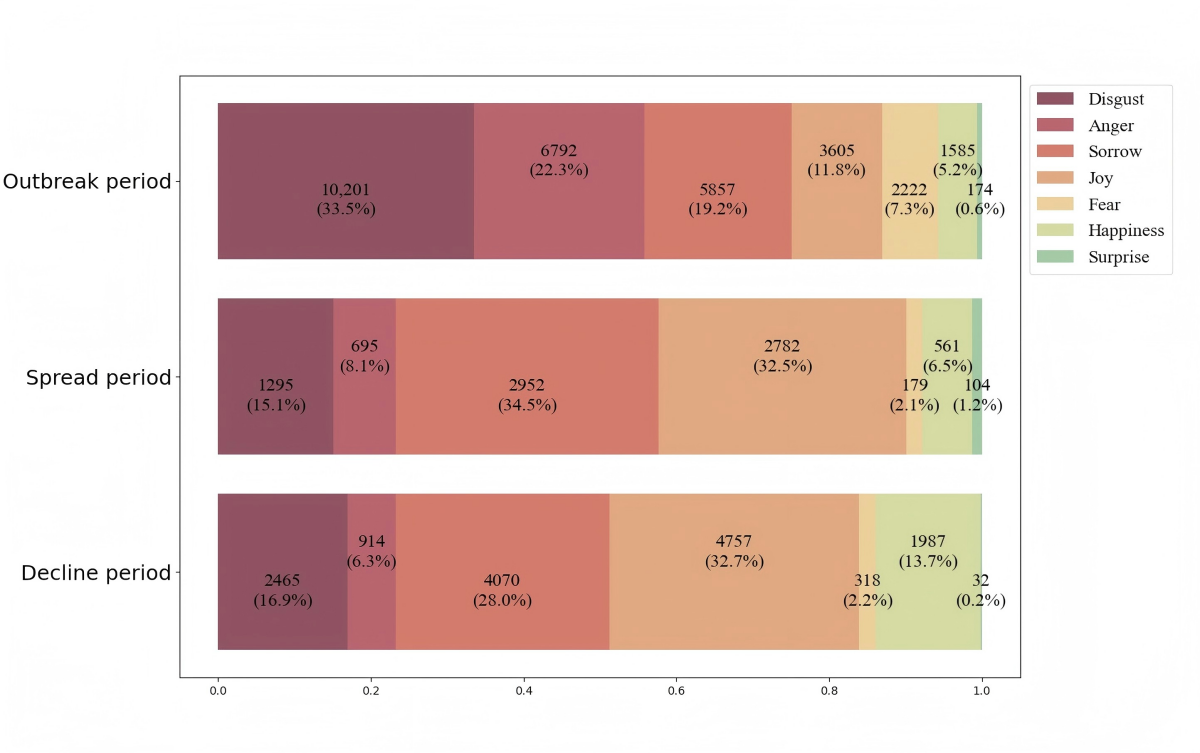
Sentiment trends in the 3 periods.

The sentiment analysis results for the 3 periods are shown in Figure 7, reflecting significant changes in public sentiment. During the Outbreak period, of the 30,436 comments, 10,201 (33.52%) expressed disgust, followed by 6792 (22.32%) showing anger and 5857 (19.24%) reflecting sorrow. In the Spread period, of the 8568 comments, 2952 (34.45%) expressed sorrow, and 2782 (32.47%) indicated joy. In the Decline period, of the 14,543 comments, 4757 (32.71%) exhibited joy, while 4070 (27.99%) conveyed sorrow and 2465 (16.95%) reflected disgust. Based on these results, the study mapped the evolution of sentiment to visualize changes in public sentiment across the 3 periods.

## Discussion

### Principal Findings

In this study, data from Sina Weibo were analyzed to investigate public sentiment during the Beijing Chaoyang Hospital assault incident using 3 analytical approaches. First, TF-IDF keyword analysis identified key terms such as “injury,” “doctor,” and “recovery,” illustrating how the initial public focus on the specifics of the incident gradually expanded to include broader health care issues. Second, thematic analysis via the LDA topic model revealed that public attention shifted from immediate responses to long-term implications, reflecting an evolution from shock to systemic consideration. Last, sentiment analysis using a Chinese sentiment lexicon indicated that negative sentiment was sustained despite progressing toward recovery-focused dialogue. Collectively, these findings revealed that negative sentiment persisted even as public dialogue progressed toward recovery-focused themes, underscoring the need for communication strategies responsive to immediate and evolving public sentiments in health-related crises.

### TF-IDF Keyword Analysis

The keyword analysis through TF-IDF conducted during the Beijing Chaoyang Hospital assault incident revealed a nuanced evolution in public sentiment, corroborating findings from previous research on crisis communication and public health emergencies. During the outbreak period, keywords such as “injury,” “doctor,” and “harm” dominated the public discourse. These terms echoed sentiments typically observed in immediate responses to medical crises, aligning with studies that indicated initial public reactions were often driven by shock and a quest for immediate understanding of the incident [57,58]. As the situation progressed to the spread period, a noticeable shift occurred toward more empathetic and supportive terms such as “gentle,” “blessings,” and “kindness.” This transition from focusing on the incident’s negative aspects to fostering a healing-oriented dialogue was consistent with the “recovery phase” described in the Coombs Crisis Communication Model [59], where the public focus shifted toward rehabilitation and emotional support. By the decline period, the emphasis on “recovery” and “gentle” further confirmed the trend toward normalization and reconciliation. In this phase, the affected community began to heal and rebuild, as noted by Ogie et al [60].

Although the keyword analysis provided valuable insights into shifting public sentiment, it had limitations. Keywords alone could not fully capture the complexity of public emotions or the depth of thematic elements evolving during such incidents. Thus, this initial analysis laid the foundation for a more comprehensive exploration. The subsequent phase used thematic and sentiment analysis techniques to delve deeper into the underlying narratives and emotional contours of public discourse, offering a more complete understanding of the incident’s impact on public sentiment.

### Thematic Analysis

Upon analyzing and summarizing the LDA topic model, it became evident that the theme evolved across all 3 phases of public opinion outbreak and spread. The decline period illustrated the theme’s continuity and the distinctions between phases. This conclusion was consistent with previous public opinion research [61-63].

Initially, the public’s response was characterized by a surge in negative sentiments, focusing on the incident’s immediate impacts and demanding accountability. This intense reaction reflects the “shock phase” [59] in crisis communication theory, where the initial shock triggers a heightened emotional response from the public, demanding urgent and decisive actions. This phase often sees a spike in public engagement and outcry, evident from the prevalent themes of aggression and immediate accountability. As the crisis continued, public sentiment shifted toward a more nuanced and empathetic perspective, concentrating on the victim’s personal and professional impacts. This transition aligns with the “deliberation phase” [59] described in situational crisis communication theory, where the public starts to process the incident more profoundly and show empathy toward the victims. The moderation in tone suggests a community moving toward a collective reflection, pondering the long-term effects on those directly affected and their implications on professional practices. In the later stages, the focus shifted toward legal measures and recovery, indicating a societal shift toward resolution and systemic reflection. This phase mirrors the “resolution phase” [59], where public discourse often turns to evaluating how the crisis was handled and the steps needed to prevent future occurrences. The decreased anger and increased discussions about recovery and legal implications suggest a maturing public opinion that seeks to address systemic issues and improve doctor-patient relationships for the future.

### Sentiment Analysis

This study has highlighted the versatility and effectiveness of sentiment analysis for capturing multidimensional emotions and thematic evolution. Previous research demonstrated these methods as reliable tools for understanding public discourse and informing interventions in complex social systems [43]. Previous research had determined that public opinion on health-related topics was inconsistent and unstable. Fluctuations influenced by topic changes led to significant variations in the dominant sentiment at each stage of public opinion development [64-66]. This study, however, indicated that, in serious doctor-patient conflicts, negative sentiments from the initial stage often persisted despite potential positive developments, driven by the primacy effect—where early impressions resist change over time [67].

During the outbreak period, negative emotions like disgust and anger predominated, aligning with Le Bon’s Theory of the Crowd [68], which attributed emotional contagion to impulsive group behavior. As the crisis matured into the spread period, the emotional landscape shifted significantly toward more nuanced emotions such as sorrow and joy. This transition was explained by the cognitive dissonance theory by Festinger [69], which explains attitude adjustments to reduce discomfort from conflicting information. However, in the decline phase, negative sentiments persisted longer than typical patterns observed in public health crises. The theory of affective intelligence [70] suggests that emotional responses could override cognitive assessments, mainly when initial emotions were intense and damaging. Such emotional endurance contradicted the general expectations derived from mood management theory [71], positing that individuals would seek to alter their mood toward more favorable outcomes, mainly as situations developed and new information became available.

The findings underscore the resilience of negative sentiments in severe doctor-patient conflicts, driven by the primacy effect and amplified by structural challenges. This persistence highlights the need for tailored communication strategies in health care settings, addressing the long-term impact of initial negative impressions and prioritizing transparent, empathetic engagement to shift public sentiment effectively.

Recent research has highlighted the importance of crisis communication in shaping public sentiment during health emergencies [72]. Clear messaging has been shown to reduce negative sentiments in the early stages, whereas delayed communication has exacerbated distrust and fear, prolonging negativity [73]. Furthermore, the cultural context plays a significant role, with collectivist societies experiencing more enduring negative sentiments due to heightened communal sharing of emotions [74]. These findings align with this study’s observations.

Comparatively, sentiment trends in other countries revealed both similarities and differences. Globally, negative sentiment dominates during crisis outbreaks, but western countries often transition more quickly to themes of systemic reform and empathy [75,76]. In contrast, distrust and fear persisted longer in China, influenced by health care resource disparities and overcrowding [77]. These findings underscore the cultural and systemic factors shaping sentiment dynamics and highlight the importance of communication strategies tailored to local contexts.

### Conclusion

Weibo, China’s largest social media platform, provides a unique view of public sentiment through its rich emotional content. By analyzing these data, researchers can identify trends in public sentiment, aiding government and relevant departments with developing effective communication strategies for doctor-patient conflicts. The study found that public sentiment evolved through different phases of public opinion, showing predominantly negative emotions during the outbreak phase, balanced sentiments during the spread phase, and continued negative dominance in the decline phase. This finding differs from previous studies, highlighting the persistent impact of initial emotions throughout the public opinion life cycle.

To address this persistence, medical institutions should implement tailored communication strategies. Establishing dedicated crisis communication teams trained in empathetic and transparent messaging can mitigate the long-term impact of negative sentiments. Timely information dissemination through clear protocols and community-based platforms can help reduce misinformation and foster public trust. Real-time sentiment monitoring systems can provide insights into public emotions as they evolve, guiding adaptive strategies to address emerging concerns effectively.

Furthermore, the methodologies used in this study, particularly the integration of sentiment lexicon analysis and LDA topic modeling, hold potential for broader adaptation across sociocultural contexts. Adapting the sentiment lexicon for other linguistic settings could enhance global sentiment analysis accuracy. Additionally, interdisciplinary applications of these findings—spanning health care management, psychology, and public health—could foster a deeper understanding of societal sentiments and their impact on health care practices. These insights can inform the development of targeted policies to improve communication strategies, train health care professionals in emotional intelligence, and enhance trust in health care systems, ultimately improving patient satisfaction and crisis management outcomes.

Future research could explore comparative studies across diverse health care systems and integrate multimodal data sources, such as images and videos, to provide a more comprehensive understanding of public sentiment dynamics.

### Limitations

Although the study presented some innovative findings, it still has certain limitations. First, the data were obtained exclusively from Sina Weibo, which, although a leading social media platform in China, did not represent the full spectrum of social media users across different platforms like WeChat and Douyin.

This reliance on a single platform may have introduced platform-specific biases, as its user base and communication dynamics differ from those on other platforms, potentially affecting the representativeness of the findings.

Second, the study’s reliance on textual data may have overlooked significant emotional and contextual information conveyed through images and videos, suggesting a need for multimodal data analysis. Furthermore, the sentiment analysis tools used might not have fully captured the nuances of sarcasm, irony, or regional dialects, leading to possible misinterpretations.

Future research can consider more diverse social media platforms and incorporate cross-cultural comparative studies on public sentiment in doctor-patient conflicts to understand the differences in public reaction patterns and emotional expressions across various cultural backgrounds. Incorporating multimodal data, such as visual and audio content, could enhance the richness and accuracy of sentiment analysis. Additionally, combining cross-platform data would allow researchers to compare public sentiment across different platforms and user demographics, providing a broader perspective.

Additionally, integrating social network analysis methods can provide a deeper analysis of the network structure and influential nodes of information dissemination, revealing opinion leaders and dissemination paths in doctor-patient conflict incidents. Longitudinal data collection and analysis would further enable the capture of temporal dynamics, offering insights into how sentiments evolve over time during such conflicts.

## Abbreviations

DUTIR: Information Retrieval Laboratory at Dalian University of Technology
LDA: latent Dirichlet allocation
TF-IDF: term frequency-inverse document frequency
WHO: World Health Organization
